# Prediction and Elimination of Physiological Tremor During Control of Teleoperated Robot Based on Deep Learning

**DOI:** 10.3390/s24227359

**Published:** 2024-11-18

**Authors:** Juntao Chen, Zhiqing Zhang, Wei Guan, Xinxin Cao, Ke Liang

**Affiliations:** 1 College of Mechanical Engineering, Guangxi University, Nanning 530004, China; 2 College of Mechanical and Automotive Engineering, Guangxi University of Science and Technology, Liuzhou 545000, China; 3 Guangxi Key Laboratory of Manufacturing System & Advanced Manufacturing Technology, School of Mechanical Engineering, Guangxi University, Nanning 530004, China

**Keywords:** control accuracy, EEMD-IWOA-LSTM, physiological tremor, teleoperated robot

## Abstract

Currently, teleoperated robots, with the operator’s input, can fully perceive unknown factors in a complex environment and have strong environmental interaction and perception abilities. However, physiological tremors in the human hand can seriously affect the accuracy of processes that require high-precision control. Therefore, this paper proposes an EEMD-IWOA-LSTM model, which can decompose the physiological tremor of the hand into several intrinsic modal components (IMF) by using the EEMD decomposition strategy and convert the complex nonlinear and non-stationary physiological tremor curve of the human hand into multiple simple sequences. An LSTM neural network is used to build a prediction model for each (IMF) component, and an IWOA is proposed to optimize the model, thereby improving the prediction accuracy of the physiological tremor and eliminating it. At the same time, the prediction results of this model are compared with those of different models, and the results of EEMD-IWOA-LSTM presented in this study show obvious superior performance. In the two examples, the MSE of the prediction model proposed are 0.1148 and 0.00623, respectively. The defibrillation model proposed in this study can effectively eliminate the physiological tremor of the human hand during teleoperation and improve the control accuracy of the robot during teleoperation.

## 1. Introduction

Because of their high precision, good flexibility, and adaptability, teleoperated robots have been widely used in medical surgery [1], aerospace [2], underwater operations [3], and harsh work environments [4]. In particular, high-precision control in complex, dangerous, or highly specialized environments can be accomplished by teleoperated robots, which has attracted wide attention [5,6]. The accuracy, reliability, and safety of the teleoperation system can be improved by effectively predicting and eliminating the disturbance of physiological tremors. In the actual working process, the robot is required to be able to execute commands quickly and accurately [7]. In particular, in the control process of some surgical robots, the accuracy requirement is about 0.1 mm [8,9]. For this high-precision control procedure, even in normal operating procedures, the operator’s hand also has a certain degree of physiological tremor, which seriously affects the high-precision control of the secondary end [10]. Therefore, accurate prediction and elimination of tremor signals is the key to improving the motion accuracy, safety, and reliability of the teleoperation system [11].

In past studies, researchers have proposed methods for eliminating the influence of tremors. Traditional filtering methods often used Kalman filters [12], Fourier linear combiners [13], digital filtering [14], and other methods to suppress physiological tremors. Digital filters often suffer from phase lag, leading to distortion, and traditional filtering methods require designing relevant filter parameters based on prior knowledge of tremor signal characteristics to eliminate tremor. In recent years, researchers have made a series of improvements and have proposed an autoregressive model combined with a Kalman filter, which could reduce the phase delay [15]. The zero-phase adaptive fuzzy Kalman filter in [16], and the AR moving average (ARMA) model to simulate and eliminate physiological tremors collected during eye surgery in [17] are somewhat of an improvement compared with traditional models.

Although traditional methods exhibit good performance in suppressing tremors, neural networks, due to their strong learning capabilities, have been applied to tremor suppression models in robot control. In [18], a quaternion variant for the Extreme Learning Machine (QELM) was developed for multi-step prediction of tremor motion. A filter based on support vector machines was proposed in [19], and an improved recursive singular spectrum analysis estimator and an extreme learning machine predictor were proposed in [20]. In [21], an extreme learning machine with an improved equalization optimizer for extensive learning (IEO-BLELM) was proposed. However, these studies did not consider EEMD decomposition, which could effectively enhance the features of input data [22]. The EEMD decomposition strategy served to enhance data features through data reconstruction [23]. The input data was regarded as a signal containing several local frequency features, and the signal was decomposed by extracting these local frequency features step by step. Wu and Huang proposed that EEMD could effectively process nonlinear signals [24]. More and more researchers combine EEMD with neural networks for feature enhancement. Song et al. [25] used the combination of EEMD decomposition and neural network models to predict nonlinear data. In [26], Yang et al. primarily combined various neural networks with EEMD decomposition to predict time series data, with results indicating the suitability of EEMD for predicting complex time series signals. It was found that combining the EEMD decomposition feature enhancement strategy with an artificial neural network could effectively improve the performance of the model. In this study, the EEMD decomposition strategy was introduced to enhance the data characteristics, and the structure of the LSTM model was redesigned according to the characteristics of the tremor signal. The predictive performance of the EEMD-LSTM model designed in this paper is closely related to the number of iterations, the number of hidden layer neurons, the learning rate, and the regularization parameters of the model. Selecting suitable parameters for the EEMD-LSTM model is the key to improving the performance of combined prediction model [27].

Yang et al. [28] proposed an improved IWOA optimization algorithm to adjust the hyperparameters of the LSTM model. The prediction performance of the model can be fully utilized with appropriate hyperparameters [29,30]. Compared with genetic algorithms, simulated annealing, gray Wolf optimization. And other algorithms [31,32,33], WOA has global search diversity, fast convergence, and good robustness [34]. Therefore, this study’s improved fusion IWOA algorithm was proposed to optimize the number of iterations, the learning rate, the number of hidden layer neurons, and the regularization parameters of the EEMD-LSTM model designed in this paper.

The main contribution of this paper is to develop a high-precision Tremor Suppression Model (TSM) to eliminate the errors caused by physiological tremors in the teleoperation control system. In addition, this paper proposes a new deep learning neural network prediction model, an LSTM Neural Network Combined with IWOA Based on EEMD. The EEMD feature enhancement method and optimization algorithm are combined with the neural network model to predict the tremor signal, and the TSM model is used to eliminate the predicted tremor signal and get the predicted expected signal so as to restore the original working signal to the maximum extent and improve the control accuracy of the teleoperated robot.

The contents of this paper are as follows: The first part summarizes the previous research and introduces the innovations of this research. In Section 2, the control flow of a teleoperated robot, the mathematical model of tremor suppression and the evaluation index of model performance are introduced. Section 3 introduces the network architecture of the prediction model design and the process of building the prediction model in this study. In Section 4, the prediction results of the model are compared and analyzed. Section 5 summarizes the conclusions of this paper and future work.

## 2. Teleoperation System

### 2.1. Teleoperation Control System

**Figure 1:**
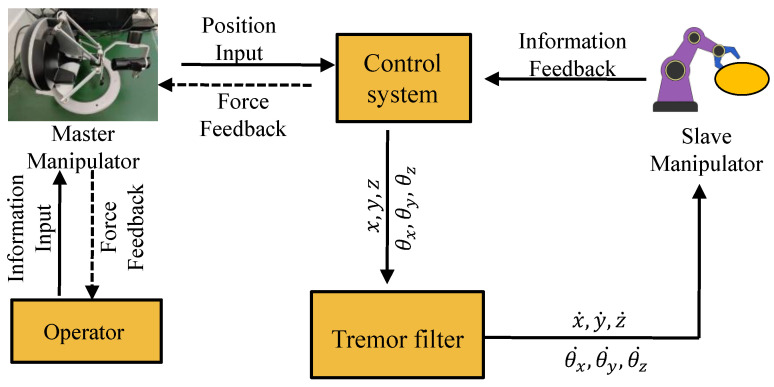
Control flow chart of teleoperation system.

Teleoperated robots mainly include four parts: operator, master manipulator, robot, and communication link [35]. According to the control process given in Figure 1. In teleoperation control requiring high-precision control, physiological tremors exist in the human hand when the operator manipulates the master manipulator. As tremors from the small portion operated by the human hand are transmitted to a larger portion of the robot, the tremor amplitude is amplified, significantly affecting the precision and accuracy of the high-precision control process [36]. Therefore, in order to improve the motion accuracy in the process of teleoperation, it is necessary to predict and eliminate the error caused by the tremor signal. Control precision can be effectively improved by using predicted pose information to control the robot. The tremor compensation framework studied in this paper requires the detection of tremor motion in the master operator, which can be obtained by various means (i.e., optical, inertial, or magnetic sensors) [37,38,39]. At the same time, the operator can get feedback from the environment and the interaction of the robot so that it can understand the work process of the robot in real time and provide information for the next operation.

### 2.2. The Mathematical Model of Tremor Suppression

The actual signal in teleoperation control includes the interference signals of hand tremors and the desired control signal.

(1) a(k)=e(k)+t(k)

t(k) and e(k) are the interference signals of hand tremors and the desired control signals, respectively.

The interference of tremor signals on robot control can be reduced by predicting and eliminating tremor signals, which can be suppressed and eliminated by the following mathematical model [40]:

(2) e′(k)=a(k)−t′(k)=e(k)+t(k)−t′(k)

e′(k) and t′(k) are predicted desired control signals and predicted interference signals of hand tremors, respectively. When t′(k)=t(k), the interference of the physiological tremor signal can be completely eliminated, and the movement of the robot can perfectly meet the desired movement.

**Figure 2:**
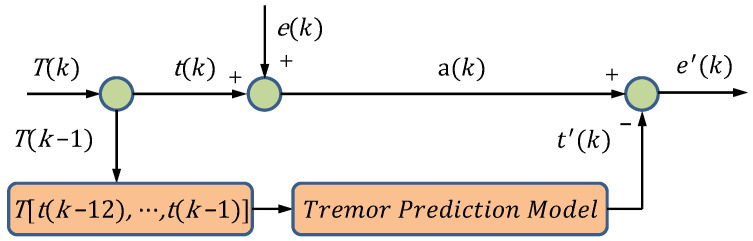
Tremor Suppression Model.

In this paper, we define a tremor suppression model of neural network for time series data. As shown in Figure 2, the TSM model predicts the next tremor signal by using the previous time series tremor signal. According to Equation (2), the actual signal is subtracted from the tremor signal predicted by the model to obtain the predicted expected signal, which is used to control the high-precision motion of the robot instead of the expected signal.

### 2.3. Performance Evaluation Indexes

In this study, regression coefficients ($R^{2}$), mean absolute error (MAE), mean square error (MSE) and symmetric mean absolute percentage error (SMAPE) were used to evaluate the prediction performance of the model, as follows:

(3) $$R^{2}=1-\frac{{\Sigma_{i=1}^{N}\left(\hat{y}\left(i\right)-y\left(i\right)\right)}^{2}}{{\Sigma_{i=1}^{N}\left(\bar{y}\left(i\right)-y\left(i\right)\right)}^{2}}$$

(4) $$MAE=\frac{1}{N}\Sigma_{i=1}^{N}\left|\hat{y}\left(i\right)-y\left(i\right)\right|$$

(5) $$MSE=\frac{1}{N}\Sigma_{i=1}^{N}{\left(\hat{y}\left(i\right)-y(i)\right)}^{2}$$

(6) $$SMAPE=\frac{1}{N}\Sigma_{i=1}^{N}\frac{\left|\hat{y}\left(i\right)-y(i)\right|}{(\left|\hat{y}\left(i\right)\right|+\left|y\left(i\right)\right|)/2}$$

## 3. Model and Method

### 3.1. LSTM Prediction Model

**Figure 3:**
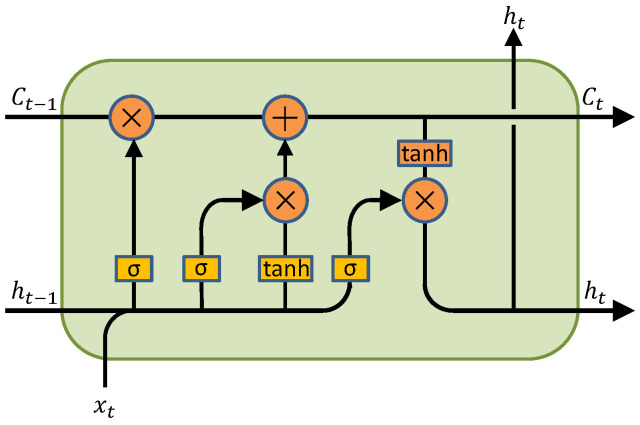
LSTM structure diagram.

LSTM is a special kind of RNN (recurrent neural network). Compared with traditional RNNS, LSTM can process and predict time series data more effectively by introducing the concepts of memory cells, input gates, output gates, and forgetting gates [41]. The model structure is shown in Figure 3.

(7) $$i_{t}=\sigma \left(W_{xi}x_{t}+W_{hi}h_{t-1}+W_{ci}c_{t-1}+b_{i}\right)$$

(8) $$f_{t}=\sigma (W_{xf}x_{t}+W_{hf}h_{t-1}+W_{cf}c_{t-1}+b_{f})$$

(9) $$c_{t}=f_{t}c_{t-1}+i_{t}\tanh\left(W_{xc}x_{t}+W_{hc}h_{t-1}+b_{c}\right)$$

(10) $$h_{t}=o_{t}tanh(c_{t})$$

where: *i* is the input gate; *f* is the oblivion door; *C* is the cell state; *o* is the output gate; *W* is the corresponding weight coefficient matrix; *b* is the corresponding offset term; σ is the sigmoid activation function; tanh is the hyperbolic tangent activation function.

### 3.2. EEMD-LSTM Prediction Model

**Figure 4:**
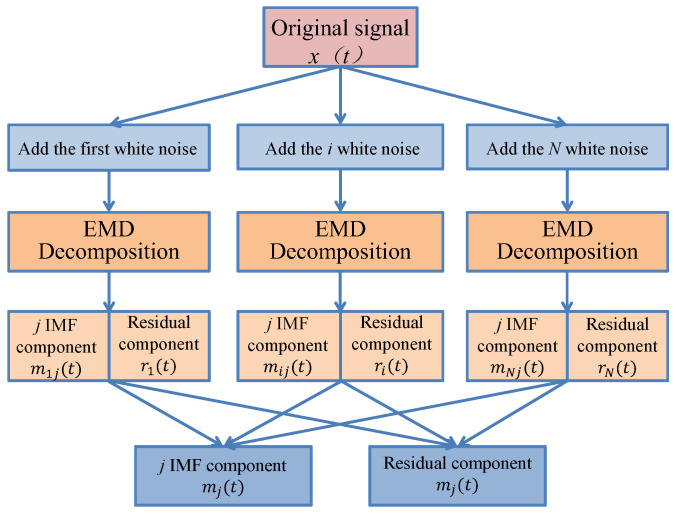
Decomposition process of EEMD.

As shown in Figure 4, EEMD is an improved EMD method, which is a very effective means to deal with nonlinear and non-stationary signals. By adding random noise to the original signal, the signal can be fully decomposed at all scales, and the influence of mode aliasing is weakened, thus improving the completeness and accuracy of the IMF. By using EEMD, the original nonlinear tremor signal can be decomposed into multiple intrinsic modal component functions (IMF) and a residual sequence (res). The decomposed results have different fluctuation characteristics, which are suitable for the prediction of time series data studied in this paper [42].

**Figure 5:**
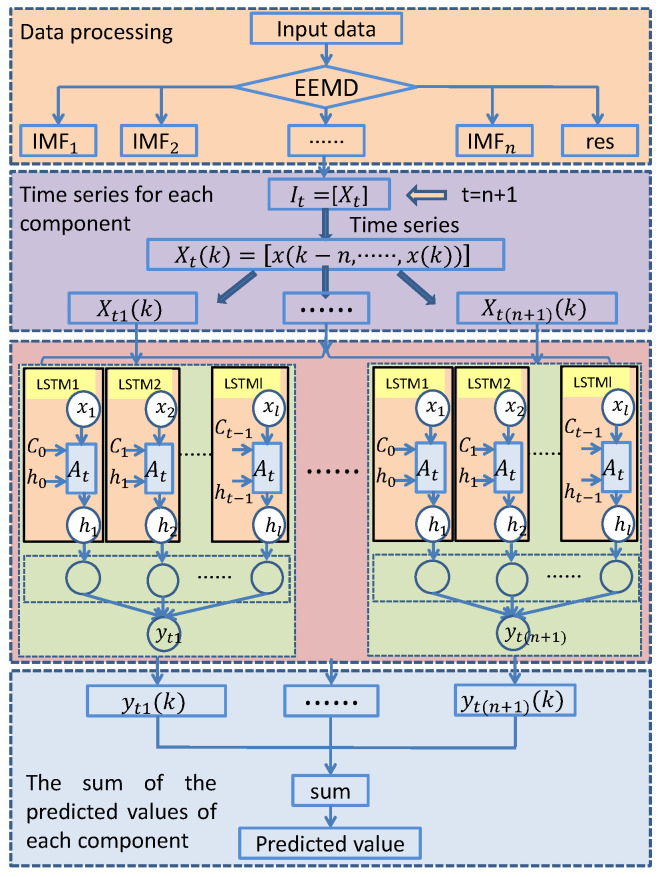
EEMD-LSTM model structure diagram.

As shown in Figure 5, the basic idea of the EEMD-LSTM algorithm is to extract the data characteristics of tremor data at different resolutions. The original time series was decomposed through EEMD, and then each IMF was used as the input for the LSTM, and the LSTM model was used to forecast each IMF. Each LSTM model will train and predict the time series and finally combine their prediction results to get the final prediction result. The advantage of the EEMD-LSTM algorithm is that by establishing multiple models, different initialization conditions and parameter combinations can be used to increase the diversity of models and improve the overall prediction accuracy. At the same time, EEMD can more accurately extract complex patterns in a time series, which helps to improve the accuracy of prediction [43].

### 3.3. Improved Whale Optimization Algorithm

**Figure 6:**
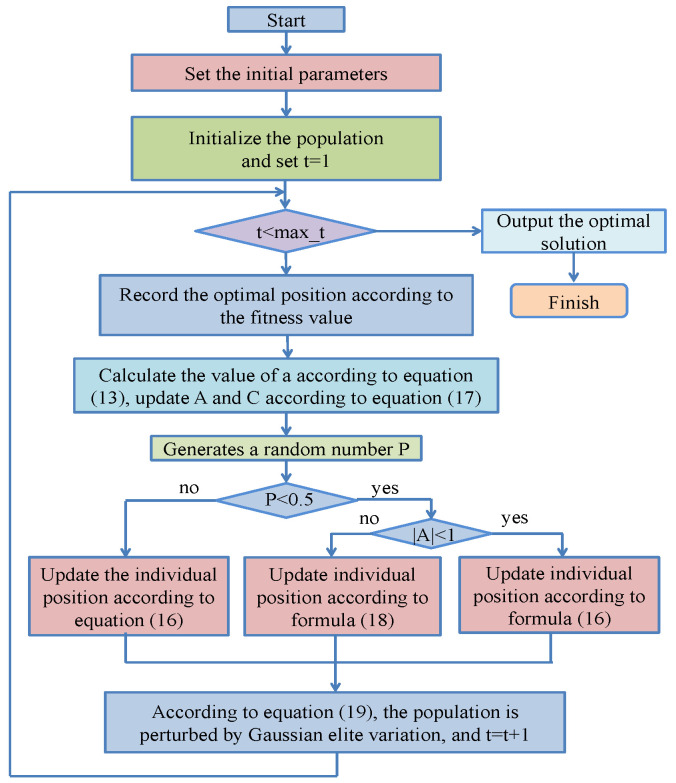
IWOA flow chart.

The Whale Optimization Algorithm (WOA) is a new swarm intelligence optimization search method. The WOA consists of three stages: search for food, shrink surround, and spiral update [44]. The whale optimization algorithm does not need to manually set various control parameter values. WOA has a novel structure and better performance in many optimization processes. As shown in Figure 6, WOA easily produces local optimal and local development ability and global search ability imbalances. By combining the advantages of different improvement methods, an improvement strategy for the WOA is proposed.

#### 3.3.1. Quasi-Reverse Learning Initializes the Population

The initialization population of an optimization algorithm has a direct impact on the optimization performance of the algorithm. However, the traditional WOA adopts the method of random population initialization, which cannot fully explore the whole search space, resulting in the degree of excellence of the algorithm being limited. In order to make the initial population more diverse, this study adopted improved reverse learning, namely quasi-reverse learning, to initialize the population [45,46]. Suppose the size of the whale population is N, $x_{i}^{j}$ is the position of the ith whale in d dimension, and ${\hat{x}}_{i}^{j}$ is its corresponding inverse solution.

(11) $${\hat{x}}_{i}^{j}=a_{i}^{j}+b_{i}^{j}-x_{i}^{j}$$

$a_{i}^{j},b_{i}^{j}$ represent the upper and lower bounds of $x_{i}^{j}$, respectively, and their corresponding quasi-inverse solutions are as follows.

(12) $$\hat{Q}x_{i}^{j}=\left\{\;\begin{array}{c} M_{i,j}+({\hat{x}}_{i}^{j}-M_{i,j})\times rand(0,1);\;\;x_{i}^{j}<M_{i,j}\\ {\hat{x}}_{i}^{j}+(M_{i,j}-M_{i,j})\times rand(0,1);\;\;x_{i}^{j}\geq M_{i,j} \end{array}\right.$$

where, $M_{i,j}=(a_{i}^{j}+b_{i}^{j})/2;$

$\hat{Q}x_{i}^{j}$ is the quasi-reverse solution corresponding to $x_{i}^{j}$. The initial populations are combined by randomly generated N initial populations and quasi-reverse learning and are sorted according to fitness. Then, the first N terms are selected as the initial populations in this paper.

#### 3.3.2. Nonlinear Convergence Factor

In the traditional WOA, the convergence factor is a linear function that controls the convergence rate of the optimization algorithm. However, the linear convergence factor often causes the algorithm to fall into a local optimal solution and lacks a global search ability [47]. Therefore, a nonlinear convergence factor is proposed to solve the above problems. It can better exert the local search and global search abilities of the algorithm.

(13) $$a=2-2\sqrt{\frac{t}{max\_t}}$$

#### 3.3.3. Adaptive Weight Strategy

The WOA is prone to falling into local optima and precocious convergence occurs during late local development. By introducing an adaptive weight strategy to improve the convergence speed and local search ability of the whale population optimization algorithm, an adaptive weight updating strategy is proposed in this paper [48].

(14) $$\omega =\left|cos\frac{\pi t}{max\_t}\right|$$

(15) $$D=|C\cdot X_{p}(t)-X(t)|$$

(16) $$X(t+1)=\left\{\begin{array}{rr} \omega \cdot X_{p}(t)-A\cdot D,\;\; & p<0.5\\ \omega \cdot X_{p}(t)+D\cdot e^{bl}\cdot cos(2\pi l),\;\; & p\geq 0.5 \end{array}\right.$$

A and C are the coefficient vectors; $X_{p}(t)$ is the current optimal solution; X(t) is the position vector of the individual; p is the random number belonging to $\left[-1,1\right]$; and *b* is the constant used to define the logarithmic spiral shape; l is a random number from $\left[-1,1\right]$. And

(17) $$A=2a\cdot r_{1}-a,C=2\cdot r_{2}$$

$r_{1}$ and $r_{2}$ are random numbers $\left[0,1\right]$, respectively.

(18) $$D=|C\cdot X_{rand}(t)-X(t)|,X(t+1)=X_{rand}(t)-A\cdot D$$

$X_{rand}(t)$ is the position vector for randomly selected whale individuals from the current population.

#### 3.3.4. Gaussian Elite Variation Strategy

The Gaussian elite mutation strategy is used to mutate the position of the globally searched whale, make full use of the characteristics of the Gaussian distribution function to generate new individuals, enhance the diversity of the group, help the algorithm improve local optimization, and improve the global search ability of the whale. The elite Gaussian mutation strategy is as follows [49]:

(19) $$X(t+1)=\left\{\begin{array}{c} X_{p}\left(t\right);\;\;\;\;\;\;\;\;\;\;\;\;\;r<0.5\\ \frac{1}{\sqrt{2\pi }\sigma }e^{-\frac{(x-X_{p}(t){)}^{2}}{2\sigma^{2}}};\;\;\;\;\;\;\;\;r\geq 0.5 \end{array}\right.$$

where *r* is the randomly generated number belonging to $\left[0,1\right]$;

(20) $$\sigma =(a_{i}^{j}-b_{i}^{j})$$

### 3.4. EEMD-IWOA-LSTM

**Figure 7:**
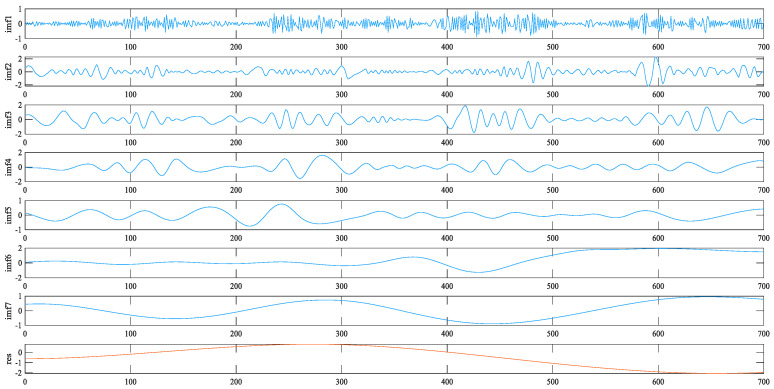
Decomposition results of EEMD.

First, the original time series is decomposed using the EEMD method to obtain multiple intrinsic mode functions (IMF). As shown in Figure 7, each component was then modeled and predicted, and the IWOA algorithm was used to optimize each IMF component prediction model to obtain the optimal model parameters. Finally, all IMF forecasts were added together to arrive at the final forecast. The advantage of the EEMD-IWOA-LSTM method is that it can fully exploit the nonlinear and non-stationary characteristics of tremor data, adapt to optimize each IMF, improve the accuracy and robustness of prediction, and be applied to various time series prediction problems. Each component obtained by EEMD decomposition can describe the fluctuation characteristics of different time scales, and the fluctuation frequencies of each component are balanced without obvious differences in time scales, effectively overcoming the mode aliasing phenomenon associated with EMD decomposition [50].

In order to accurately predict hand tremor time series data with strong nonlinear and stochastic characteristics, this paper combined the respective advantages of adaptive EEMD and LSTM neural networks. In order to give full play to the predictive performance of the model, this study proposed an IWOA algorithm to optimize the network iteration times, the number of hidden layer neurons, the learning rate, and the regularization parameters. A combined prediction model of human hand tremor time series data based on EEMD-IWOA-LSTM was designed. After establishing the EEMD-LSTM model, the component signals decomposed from EEMD are respectively input into the LSTM and optimized using IWOA, and the prediction model is established. The specific steps are as follows:

The influence of an asynchronously long input layer on the prediction accuracy of LSTM is discussed. The network architecture of LSTM is redesigned to have an input data step size of 12 and an activation function layer is added. The original input data was changed into each IMF component, residuals (res) were obtained by EEMD decomposition, and each component and residual data were modeled and predicted, respectively. The input step size of different time series data will affect the prediction accuracy of the model. In this study, the appropriate input step size is obtained by the trial-and-error method. The IWOA proposed in this study was used to optimize the number of hidden layer neurons, the iteration times, the learning rate, and the regularization parameters of the EEMD-LSTM model. Then, the influence of different activation functions on the prediction accuracy of the EEMD-IWOA-LSTM model is tested. In the process of testing the EEMD-IWOA-LSTM model proposed in this paper, two different data sets are used to test the model, and the evaluation indexes of the other models are compared. The results show that the performance of the EEMD-IWOA-LSTM model is superior to other models.

## 4. Results and Discussions

### 4.1. Results of Example 1

**Figure 8:**
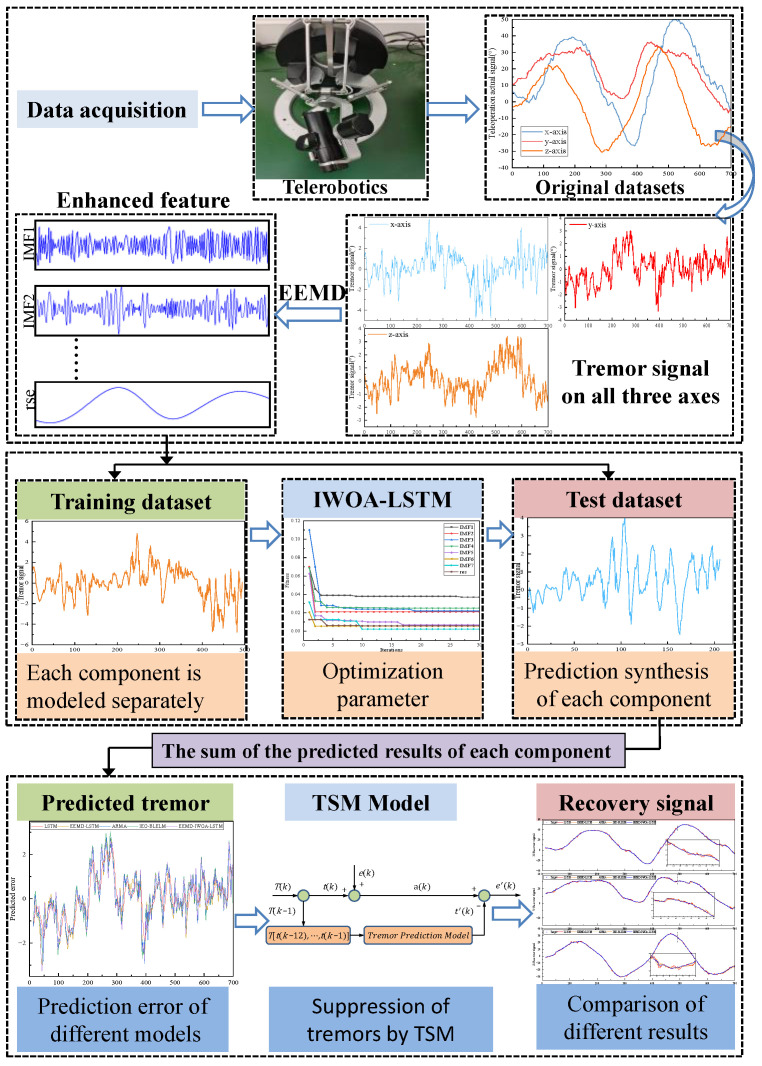
Modeling process in Example 1.

The structure of the model constructed in this study is shown in Figure 8. It is divided into three parts, the source of the original data, the decomposition of the original data, the use of EEMD to extract the fluctuation rules of different time scales in the tremor data, and the construction of the EEMD-IWOA-LSTM time series prediction model. In this study, the data used came from the remote control system described in the cited reference [51]. The data acquisition device is a high-precision data glove, the data glove can obtain a high-precision actual signal, and the physiological tremor signal of the operator’s hand can be observed in the obtained actual signal. The data used in this study are 700 sets of 3D tremor data samples collected, 70% of which are used as the training set and the remaining 30% as the test set for model construction. Different activation functions are used in the EEMD-IWOA-LSTM model to compare their effects on the prediction accuracy, and the optimal enhancement strategy is combined with EEMD-IWOA-LSTM to construct the prediction model in this paper. The step size of the input data of the time series determined by trial-and-error is 12.

The input data:

(21) X=[x1 (k−12), …, x1 (k)]

The output data:

(22) Y=[y1 (k)]

**Figure 9:**
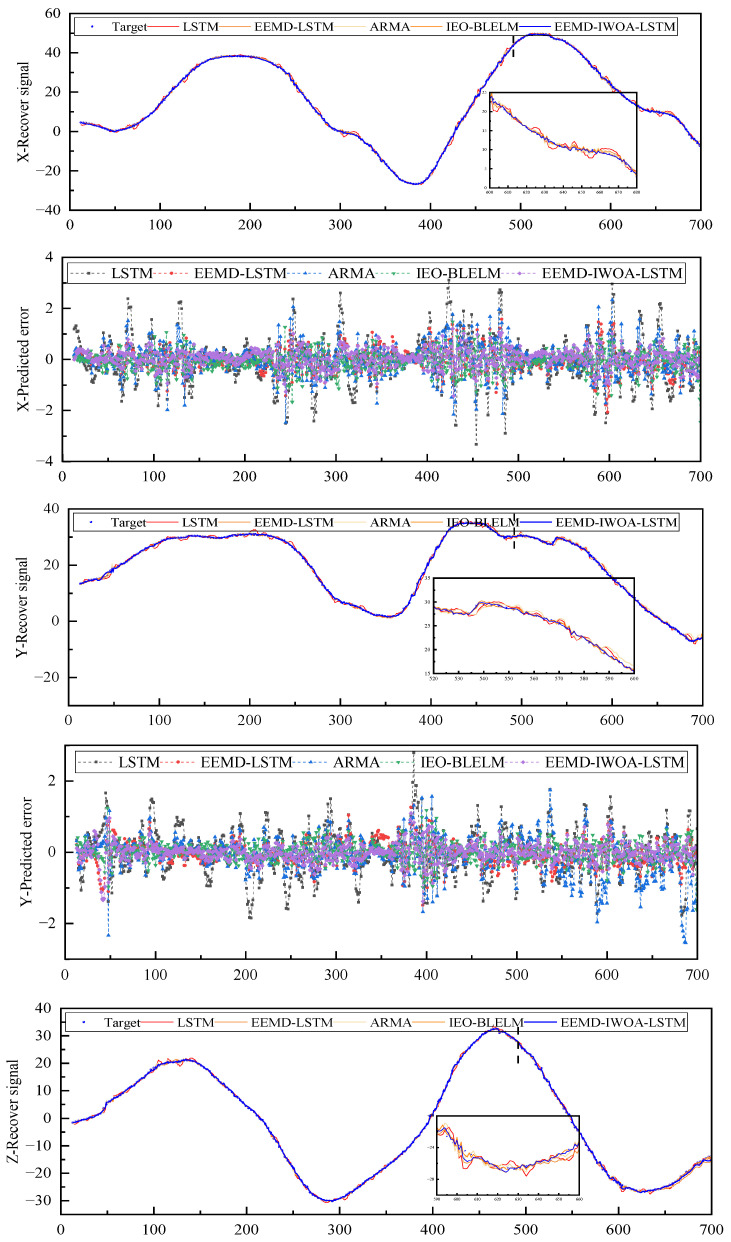

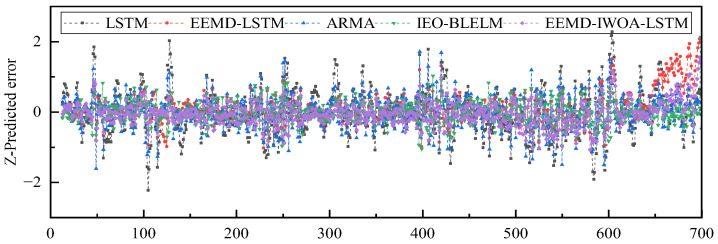
Prediction results of tremor signal.

In this study, it was found through trial and error that the best effect was to set the LSTM step size to 12, and the EEMD-LSTM model and LSTM adopted the same number of steps. As can be seen from Figure 9, the recovery signal obtained by the EEMD-IWOA-LSTM prediction model proposed in this study has the best coincidence with the actual expected signal value, and this model can effectively predict and eliminate the adverse effects of physical tremor of the hand during the control of the teleoperated robot. Compared with other models, the EEMD-IWOA-LSTM model has the highest prediction accuracy and stability for nonlinear tremor data.

As can be seen from Table 1, comparing the comprehensive prediction performance of the triaxial tremor data in Case 1, MSE, MAE, and SMAPE of the EEMD-IWOA-LSTM prediction model proposed in this paper are 0.1148, 0.2628, and 0.4934, respectively. According to the results, it can be seen that the EEMD-IWOA-LSTM model has the highest prediction accuracy, and the prediction accuracy of the EEMD-IWOA-LSTM model is obviously better than that of the traditional LSTM model. The results show that the traditional LSTM model has the largest error and poor stability in predicting the peak signal of the physiological tremor signal. The sensitivity of the EEMD-IWOA-LSTM model to signal changes at the peak of tremor data is improved by introducing suitable feature enhancement strategies and model optimization methods, which proves that feature enhancement strategies and improved optimization methods can help improve the robustness and accuracy of the model.

**Figure 10:**
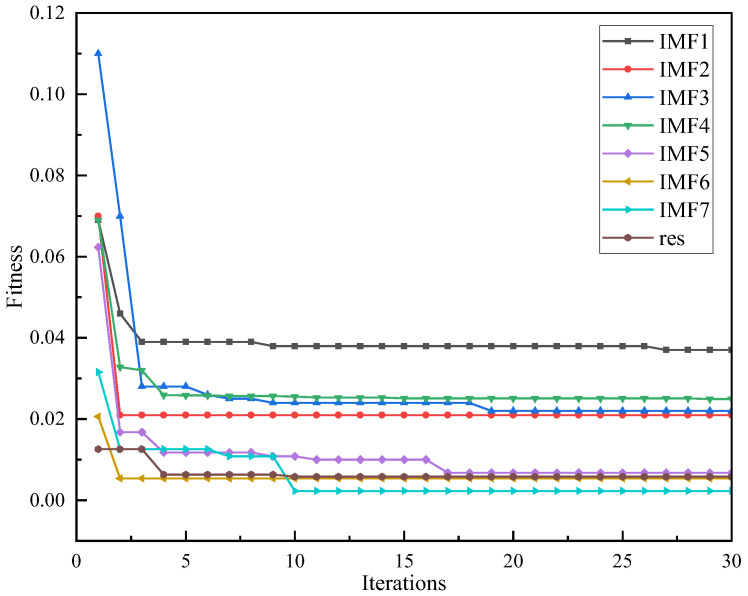
Fitness curve of each IMF component.

This is because the performance and prediction accuracy of the LSTM model are largely affected by the configuration of its hyperparameters, including but not limited to the number of iterations, the number of hidden layer neurons, the learning rate, and regularization parameters. Traditional hyperparameter adjustment methods often rely on experience and trial and error, which is not only time-consuming but also lacks systematicity, which make it difficult to ensure the optimization of model performance. In this study, the introduction of IWOA optimization can adaptively find suitable hyperparameters and give full play to the performance of the model, which shows the importance of optimizing the configuration of hyperparameters. The fitness change curve of the model is shown in Figure 10.

As shown in Figure 10, taking the optimization process of the *X*-axis in Case 1 as an example, it can be clearly seen from the figure that the fitness of each component, namely the MSE, converges quickly and decreases continuously. The number of iterations selected in this study is 30, and when the iterative optimization reaches the 20th iteration, the root-mean-square error of each IMF and res component decreases significantly and tends to be stable compared with that before optimization. Again, we highlight the importance of the optimization process.

**Figure 11:**
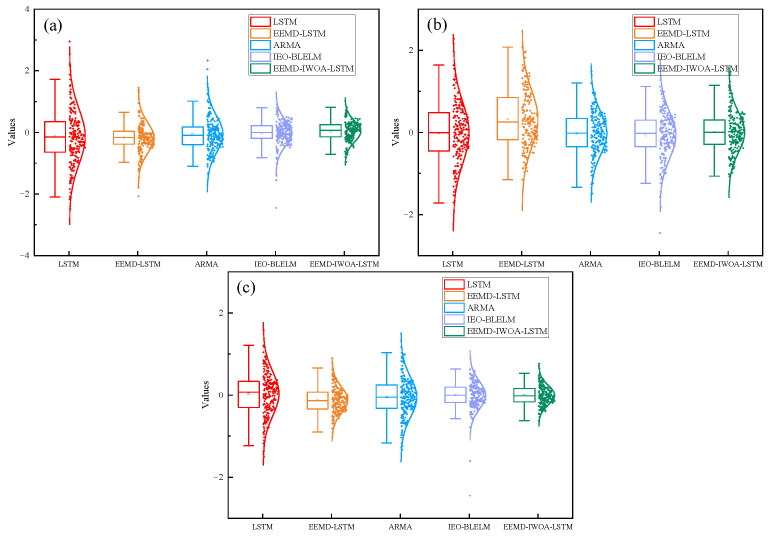
Box diagram of different axes. (**a**) is the *x* axis, (**b**) is the *y* axis, and (**c**) is the *z* axis.

As shown in Figure 11, the box plot of the prediction errors of the three axes shows that the error range of the EEMD-IWOA-LSTM model is smaller than that of the other models, and the mean value of the normal distribution is closer to zero and the variance is smaller. The EEMD-IWOA-LSTM model has the highest prediction accuracy and stability for nonlinear tremor data.

**Figure 12:**
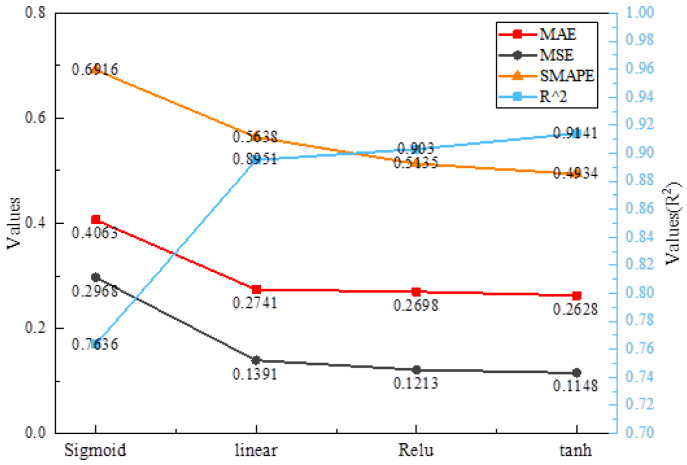
Comparison of the effects of different activation functions.

The sigmoid, linear, RELU and tanh activation functions are, respectively, applied to the EEMD-IWOA-LSTM model [52]. The result is shown in Figure 12. The prediction performance of the Sigmoid model is the worst. $R^{2}$ and MSE are 0.7636 and 0.2968, respectively. This is not enough for the prediction accuracy of nonlinear data to eliminate hand tremors during teleoperation. This is because the output of the sigmoid function is always mapped to a non-negative range, and it cannot directly produce negative output, which will reduce the sparsity of the model. When the sigmoid function approaches zero, the output value changes slowly, which causes the output value to deviate from the actual regression prediction value. In contrast, the prediction accuracy of MSE, MAE, and SMAPE increased by 61.32%, 35.32%, and 28.66%, respectively, under the action of the tanh activation function. In particular, the $R^{2}$ of the forecast increased by 16.46%. This is because the output value of the tanh activation function has a larger amplitude, helping to provide a stronger gradient signal that converges faster to the optimization goal. Secondly, the mean of the tanh function is close to zero, while the mean of the sigmoid function is close to 0.5, which can reduce the offset of the input data and help accelerate the convergence rate of the model. The numerical stability is better. When the input value is large or small, the gradient change of the tanh function is relatively moderate [52]. In contrast, the gradient change of the sigmoid function is steeper near 0 or 1, which is prone to the problem of gradient disappearance or explosion.

### 4.2. The Result of Example 2

In Example 2, the original data set is derived from the physiological tremor data of the human hand measured by the jumping motion device designed in paper [53]. The paper has the data set in the URL: https://www.kaggle.com/hakmesyo/hand-tremor-dataset-for-biometric-recognition accessed on 16 December 2022. In addition, there were a total of 5 subjects’ 3D hand motion tremor data in the data set, and this study used the hand tremor data of the first subject for prediction. In this study, the EEMD-IWOA-LSTM model proposed in Example 1 was used to predict 3D tremor signals, and the prediction performance of the model for nonlinear physiological tremor data was evaluated. 960 sets of samples were extracted from the data set, of which 70% were training sets and the rest were test sets.

**Figure 13:**
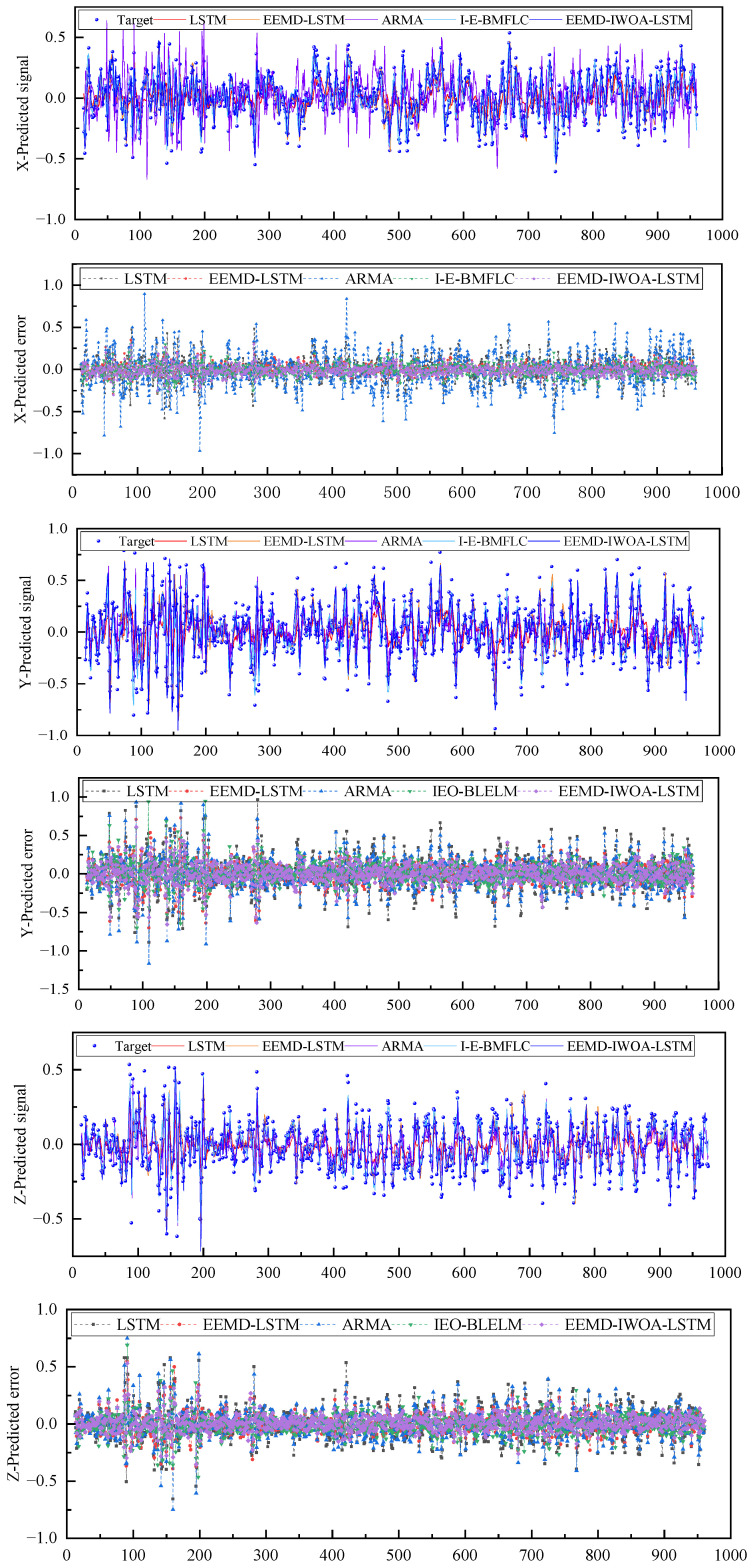
Prediction results of tremor signal.

As can be seen from the prediction results in Figure 13 regarding the physiological tremor data of the human hand in Example 2, the prediction performance of the proposed EEMD-IWOA-LSTM in this study is significantly better than that of other models, and the evaluation indicators of the EEMD-IWOA-LSTM show the same trend as those in Example 1. EEMD-IWOA-LSTM has the highest prediction accuracy, the highest degree of fitting to the original signal, and the smallest prediction error.

**Figure 14:**
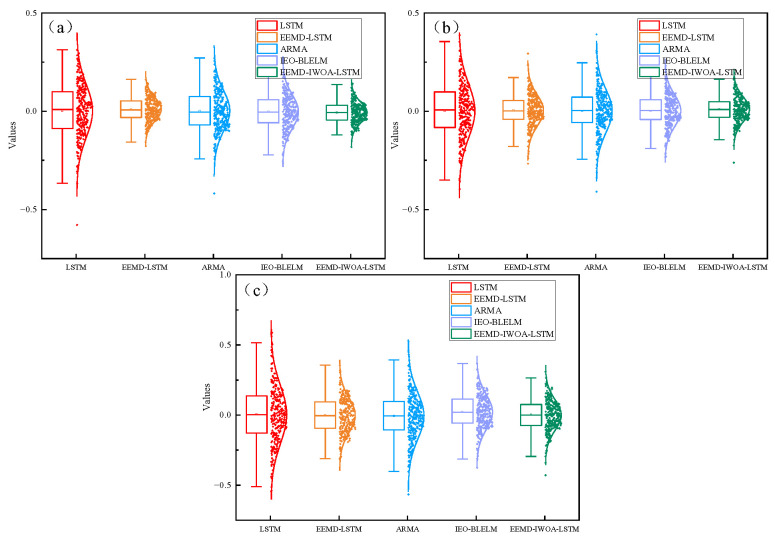
Error box diagram; (**a**) is the *x* axis, (**b**) is the *y* axis, and (**c**) is the *z* axis.

As shown in Figure 14, the box plot of the prediction errors of the three axes in case 2 shows the same trend as that in case 1. It can be seen that the quartile error range of the EEMD-IWOA-LSTM model is smaller than the other two models, the mean value of the normal distribution is closer to 0, and the variance is smaller.

As can be seen from Table 2, the EEMD-IWOA-LSTM prediction model proposed in this paper is significantly superior to other models in case 2, especially compared with EEMD-LSTM, ARMA, and IEO-LSTM. MSE decreased by 24.39%, 62.87%, and 34.04%, respectively. The $R^{2}$ increases by 6.58%, 36.10%, and 14.28%, which again shows the validity of the prediction model proposed in this paper.

**Figure 15:**
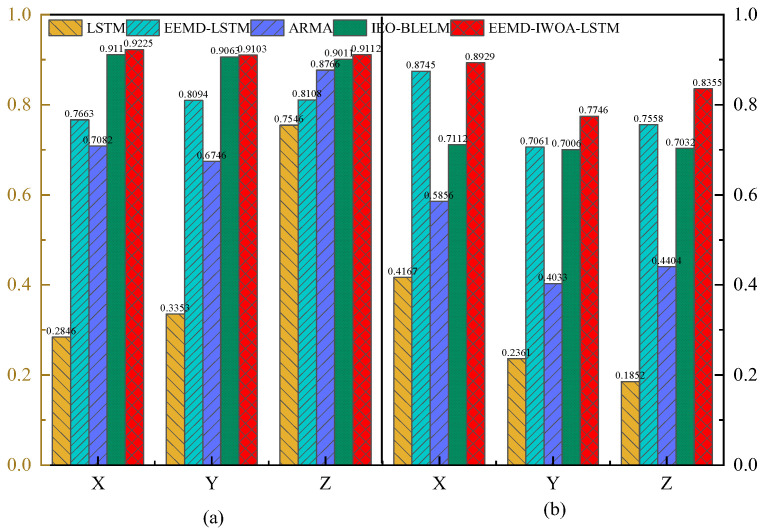
Tremor data $R^{2}$ for three axes in two cases; (**a**) Case 1; (**b**) Case 2.

As shown in Figure 15, in the two cases, the $R^{2}$ of the EEMD-IWOA-LSTM model test set proposed in this study is significantly higher than the traditional LSTM model and the EEMD-LSTM model. The predicted $R^{2}$ of the single-dimensional tremor data in both cases is not less than 0.7746, and the predicted $R^{2}$ of the three-dimensional tremor data for a single tester is not less than 0.8238. It indicates that the prediction model proposed in this study can effectively predict and reduce the influence of physiological tremors in the human hand on the high-precision control of a teleoperated robot.

## 5. Conclusions

Predicting and eliminating the physiological tremor error of the operator’s hand is a key problem for improving the control accuracy of robots during teleoperation. In order to eliminate the adverse effects of physical tremor signals and improve the accuracy and stability of teleoperation control, a mathematical model for the suppression and elimination of tremor signals in time series (TSM) was defined. A new EEMD-IWOA-LSTM time series prediction model is designed to predict nonlinear tremor signals. TSM is used to eliminate the predicted tremor signal and improve the control accuracy. Based on LSTM’s traditional time series prediction model, we explore the feature enhancement strategy for nonlinear data, decompose the original data by EEMD, and redesign the network structure of the deep learning prediction model. In order to improve the prediction accuracy of the EEMD-LSTM model for tremor data, an IWOA algorithm is designed to optimize the hyperparameters of the deep learning prediction model. The EEMD-IWOA-LSTM model proposed in this study was applied to the prediction of two different tremor data. The conclusions are as follows:

This study proposes an effective predictive model aimed at eliminating the negative effects of physiological hand tremors on the control process of remote-controlled robots. In the two examples in this paper, the MAE, MSE, and SMAPE of the model proposed in this study are significantly lower than those of the traditional model, and the $R^{2}$ is higher than 0.8238. It indicates that the EEMD-IWOA-LSTM model constructed in this study can accurately predict the tremor signals, and the predicted recovery signals can be obtained according to tremor signals that can be effectively eliminated by the TSM model. The results show that the recovery signal predicted by the model overlapped well with the actual expected signal, indicating that the model can effectively improve the control precision of the robot.

The prediction accuracy of the EEMD-IWOA-LSTM model proposed in this study is better than that of the LSTM, EEMD-LSTM, and EEMD-SSA-LSTM models for physiological tremors. The prediction performance of the model is largely affected by the parameters. By introducing the IWOA optimization algorithm, the model’s iteration times, the number of hidden layer neurons, the learning rate, and the regularization parameters are optimized, which can give full play to the efficiency of the LSTM model and improve the model’s rate and prediction accuracy. In Example 1, the MAE of the EEMD-IWOA-LSTM model decreased by 52.07%, 33.69%, 32.06%, and 3.35% compared with the LSTM, EEMD-LSTM, ARMA, and IEO-BLELM prediction models. In case 2, there was also an obvious downward trend, by 52.05%, 15.58%, 38.75%, and 28.37%, respectively. Therefore, the prediction accuracy of this model shows good prediction performance compared with the existing models.

This study provides a way to improve the prediction accuracy of time series data of physiological tremors of the human hand. It can be seen from the results that the enhancement strategy based on EEMD and multi-step input time series data can improve the accuracy and stationarity of the artificial neural network prediction model, and the IWOA optimization algorithm can optimize the hyperparameters of the model and give full play to the performance of the model. At the same time, the prediction error of the model is mainly due to the drastic changes in the tremor data at the peak, and the peak error of the model is significantly reduced after combining the enhancement strategy.

In conclusion, the EEMD-IWOA-LSTM model proposed in this study has shown good results in the prediction error and correlation of physiological tremor data of the human hand during teleoperation control and the fitting effect between the recovered signal and the expected signal obtained by the model. In the future, we consider deploying the prediction model to the actual teleoperated robot, and consider combining the time domain and frequency domain to further improve the prediction accuracy and operation efficiency of the model.

## Figures and Tables

**Table 1:** Performance indicators in Case 1.

	MAE	MSE	SMAPE	$R^{2}$
LSTM	0.5483	0.5939	0.9690	0.5638
EEMD-LSTM	0.3963	0.2869	0.6826	0.7813
ARMA	0.3868	0.2613	0.6553	0.8010
IEO-BLELM	0.2719	0.1251	0.5139	0.9106
EEMD-IWOA-LSTM	0.2628	0.1148	0.4934	0.9141

**Table 2:** Performance indicators in Case 2.

	MAE	MSE	SMAPE	$R^{2}$
LSTM	0.1243	0.0263	1.2626	0.2546
EEMD-LSTM	0.0706	0.0082	0.7486	0.7696
ARMA	0.0973	0.0167	0.9834	0.5264
IEO-BLELM	0.0832	0.0094	0.8223	0.7062
EEMD-IWOA-LSTM	0.0596	0.0062	0.6599	0.8238

## Data Availability

The experiments’ source code and required data sets can be obtained upon request.

## Nomenclature

LSTM	Long Short-Term Memory
RNN	Recurrent Neural Network
EMD	Empirical Mode Decomposition
EEMD	Ensemble Empirical Mode Decomposition
EEMD-LSTM	LSTM neural Network Combined with EEMD
WOA	Whale Optimization Algorithm
IWOA	Improved whale optimization algorithm
EEMD-IWOA-LSTM	LSTM Neural Network Combined with IWOA Based on EEMD
MAE	Mean Absolute Error
MSE	Mean Square Error
SMAPE	Symmetric Mean Absolute Percentage Error
$R^{2}$	Regression Coefficients
